# Notes on Parasites

**Published:** 1892-06

**Authors:** C. W. Stiles

**Affiliations:** Bureau of Animal Industry, U. S. Department of Agriculture


### NOTES ON PARASITES.

BY C. W. STILES, PH.D.

8. A check-list of animal parasites of cattle, with a request to Veterinarians and Zoölogists.

The following is a list of the animal parasites of cattle so far as I have been able to trace them in veterinary and zoölogical works. Although many of them have been recorded in various parts of this country, our information in regard to their geographical distribution is still very far from being complete. In fact there are several parasites in the list which, I suspect, must be present in the United States, although I have, as yet, been unable to obtain any positive data in regard to them. It is in the hope of tracing the distribution of our cattle parasites in this country, and also in the hope of obtaining data, from responsible men, in regard to their frequency in various regions, that I publish the following list and cordially invite correspondence upon the matter with all persons interested in the subject.

Check list : Various members of the Bureau of Animal Industry (Smith, Curtice, Kilborne, Hassall, Stiles) have found all those cattle parasites which are marked with an asterisk.

PROTOZOA:—

* 1. Sarcosporidia in the heart and various other muscles.
2. Balbiania gigantea, giant sarcosporidia of the œsophagus.
3. Cytospermium (Coccidium ?) Zürnii, intestines (C. perforans ?).
4. Coccidium oviforme, liver.
5–12. Ciliated Protozoa, in the stomach.
5. Bütschlia parva.
6. B. neglecta.
7. Isotricha prostoma.
8. I. intestinalis.
9. Dasytricha ruminantium.
10. Entodinium bursa.
11. E. caudatum.
12. E. minimum.

TREMATODES:—

13. Amphistoma conicum, stomach.
14. A. crumeniferum, stomach.
15. A. tuberculatum, intestines.
16. Distoma lanceolatum, small liver-fluke.
*17. D. hepaticum, common liver-fluke.
*18. D. magnum, large liver-fluke. (Syn., Fasciola carnosa, F. americana, D. texanicum.)
19. Gynæcophorus crassus (bovis), found in the portal veins, the veins of the bladder, etc.

CESTODES (tape-worms):—

*A.* Larval stage, Cysts.
*20. Cysticercus bovis, muscles. (Tænia saginata of man.)
*21. C. tenuicollis abd. cavity. (T. marginata of dogs.)
22. Cœnurus cerebralis, brain. (T. cœnurus of dogs.)
*23. Echinococcus polymorphus, liver, lungs, etc. (Syn. E. veterinorum, etc.) (T. echinococcus of dogs.)

*B.* Adult tape-worms in the intestines.
*24. Tænia (Moniezia) expansa.
25. T. (M.) denticulata.
26. (T. (M.) fimbriata.)
27. T. (M.) alba.

28. T. (M.) Benedini.
29. T. centripunctata.
30. T. globipunctata.

NEMATODES (round worms):—

31. Ascaris vituli, intestines.
32. (A. lumbricoides, intestines.)
33. (A. megalocephala, intestines.)
34. Eustrongylus gigas, kidneys.
*35. Strongylus contortus, stomach.
*36. S. Ostertagi (S. convolutus) stomach.
*37. S. ventricosus, stomach and intestines.
*38. S. micrurus, anuerysm art., bronchial tubes, trachea.
39. S. filaria, bronchial tubes.
*40. S. pulmonalis, lungs.
*41. Œsophagostoma (Str.) inflatum, intestines.
*42. Œs. columbianum (nodular disease of the intestines.)
*43. Filaria cervina, body cavity.
44. F. (cervina ?), in the eye.
45. (F. equina, body cavity.)
*46. F. lienalis, sp. nov., in Ms., a long, thin worm in the connective tissue between the spleen and stomach.
47. F. lachrymalis, lachrymal ducts and under the eyelids.
48. F. medinensis, sub-dermal connective tissue.
*49. Myzomimus scutatus (Spiroptera scutata), œsphageal epithelium.
50. Trichina spiralis, muscles (extremely rare in muscles of cattle.)
*51. Trichocephalus affinis, intestines, cœcum.
52. Nematodum bovis tauri, eye.
53. Nematodum sp., gland lymph in capsules.

HIRUDINEA—Leeches :—

54. Haemopis (Hirudo) sanguisuga, mouth, throat, nose.

ARACHNOIDEA :—

55. Pentastomum (Linguatula) tænioides, larval stage (P. denticulatum) encysted in various organs.
*55*a*. Demodex folliculorum, *var.* bovis.
56. Sarcoptes scabiei, itch.
*57. Psoroptes communis, mange.
*58. Chorioptes symbiotes, mange.
*59. Boophilus bovis, cattle tick.
*60. Amblyomma unipunctata, Packard.

*61. Dermacentor americanus, Linné.
*62. D. occidentalis, Marx, sp. nov., Ms. (California.)
*63. Ixodes scapularis, Say.
*64. I. sp. (California.)

INSECTA :—

*A.* Larval stage, grubs.

*65. Hypoderma lineata, heel-fly, grub in the back.
66. H. bovis, grub in the back.
*67. Lucilia macellaria, screw-worm.

*B.* Adult.

*68. Haematobia serrata (horn-fly.)
*69. Stomoxys calcitrans, small biting fly.
*70. Tabanus, several species, horse-flies.
*71. Simulium pecuarum, buffalo gnat.
*72. S. miridionale, turkey gnat.
73. Sarcopsylla penetrans, the burrowing flea.
*74. Haematopinus eurysternus, the short nosed louse.
*75. H vituli, the long nosed louse.
*76. H. macrocephalus, the horse louse.
*77. Trichodectes scalaris, the biting louse.

In this check list I have purposely omitted the Texas fever parasite, since the disease it causes has already been worked up geographically by the Bureau. I have further enclosed in brackets those parasites, such as *Asc. megalocephala*, the existence of which in cattle, I have reason to doubt.

It may occur to some that the geographical distribution of parasites will agree exactly with the distribution of the host. This, however, is not the case, for the climate, the conformation of the land, and the distribution of the secondary hosts, are important factors in determining the parasitic fauna.

In case any correspondent comes across a parasite which, owing to his inability to obtain the literature on the subject, he is not able to diagnose, I shall be glad to determine the species for him if he will forward the same to the Agricultural Department. Parasites to be determined may be sent in several ways :

1. They may be packed in the organs in which they are found ; or they may be preserved by either of the following methods :

2. Wash in warm water and place in from 50 per cent. to 70 per cent. alcohol.

3. (*a*) Wash in warm water, (*b*) kill in the following solution heated to 50° C., (equal parts of 70 per cent. alcohol and a saturated aqueous solution of corrosive sublimate, to which a few drops of acetic acid have been added), allow the parasites to remain in the solution (which now can be allowed to cool) five to thirty minutes according to size, then (*c*) wash in water one to twelve hours and (*d*) place in 50 per cent. to 70 per cent alcohol.

9. Two cases of *Echinococcus multilocularis* in cattle.

*E. multilocularis* (alveolaris) is a peculiar form of growth of the hydatid larval stage of *Tænia echinococcus* of the dog, in which the hydatid resembles a colloid cancer so closely that for many years the true nature of the *E. multilocularis* was not understood, until Virchow (1854) recognized in it the presence of the hydatids. Another form, *E. racemosus*, is very similar, in fact Leuckart makes it identical with *E. multilocularis.* The parasite varies in size from that of a duck's egg (Leuckart) to twice the size of a man's head (Griesinger). It has been found about seventy times in man, chiefly in Switzerland and South Germany, and has been reported in cattle by Friedreich, Huber, Perroncito, Harms, Bollinger, Brinsteiner (one case each) and Leuckart.*

I can now add two more cases of its occurrence in cattle. In one case, which I found at the Leipzig (Germany) slaughter house, the parasite was as large as a baby's head. The second case was in an American animal; only a portion of the specimen came into my hands, so I cannot state how large it was. This last case is interesting from the fact that for some time there was considerable doubt as to whether I had a case of heavy infection with a large number of small cysts, or an *E. multilocularis* of the *racemose* type, but upon closer examination I found canals connecting several small cysts, which of course, removed the doubt as to the diagnosis. So far as I can learn, this is the first case of *E. multilocularis* reported in cattle in the United States.

BUREAU OF ANIMAL INDUSTRY, U. S. DEPARTMENT OF AGRICULTURE, Feb. 3, 1892.

* Since writing the above, an article has appeared in a German journal in which it is stated that this variety is much more frequent in cattle than has heretofore been supposed.

